# 1,2,5,10,11,14-Hexaoxadispiro[5.2.5.2]hexadecanes: Novel Spirofused Bis-Trioxane Peroxides

**DOI:** 10.3390/molecules13081743

**Published:** 2008-08-19

**Authors:** Axel G. Griesbeck, Lars-Oliver Höinck, Johann Lex, Jörg Neudörfl, Dirk Blunk, Tamer T. El-Idreesy

**Affiliations:** 1 Department of Chemistry, Institute of Organic Chemistry, Greinstr. 4, 50939 Köln, Germany; E-mail: griesbeck@uni-koeln.de; 2 Department of Chemistry, Faculty of Science, Cairo University, Giza, Egypt; E-mail: tamertawhid@yahoo.com

**Keywords:** Bis-spiro compounds, peroxides, singlet oxygen, DFT calculations, X-ray structure determination

## Abstract

A set of new bis-spirofused 1,2,4-trioxanes **4a**-**d** was obtained from the reaction of cyclohexane-1,4-dione with allylic hydroperoxides **2a**-**d**, bearing an additional hydroxy group in the homoallylic position, by diastereoselective photooxygenation of allylic alcohols **1a**-**d** and subsequent BF_3_-catalyzed peroxyacetalization with the diketone. From the reaction of a monoprotected cyclohexane-1,4-dione **5** with the allylic hydroperoxide **6** derived from the singlet oxygenation of methyl hydroxytiglate, one monospiro compound was obtained, the 1,2,4-trioxane ketone **7**, as well as a mixture of the diastereoisomeric *syn*- and *anti* bis-1,2,4-trioxanes **8**. The structures of bis-1,2,4-trioxanes were examined theoretically by DFT methods and compared with X-ray structural data in order to evaluate the preferential trioxane ring conformational orientation.

## Introduction

Artemisinin (qinghaosu) is a prominent tetracyclic sesquiterpene peroxide isolated from the leaves of *Artemisia annua* [1]. Due to its remarkable antimalarial activities at the nanomolar level, artemisinin, its derivatives, and several analogs have become important as antimalarial drugs having the highest activities against multidrug-resistant forms of *Plasmodium falciparum* [2]. The high pharmacological potential of artemisinin, combined with its synthetically challenging structure have prompted Hofheinz [3], Zho [4], Avery [5], Liu [6], Yadav [7] and others to carry out total syntheses of this target and of structurally related compounds. There is an ongoing debate on whether or not the chemical reactivity of artemisinin derivatives can be directly correlated to the biological activity and what kind of peroxide-related steps actually interfere with parasite-infected cells [8]. The cyclic peroxide moiety is the central pharmacophore in artemisinin with the 1,2,4-trioxane group being a part of a bicyclo[3.2.2]nonane skeleton. A structural simplification which has been extensively realized synthetically and examined with respect to antimalaria-activity is the 3-spiroannelated 1,2,4-trioxane structure [9]. We have recently started to explore synthetic routes to these compounds as well as to ring-contracted bicyclic peroxides with intact 1,2,4-trioxane units as well as to multifunctional trioxanes [10].

**Scheme 1:**
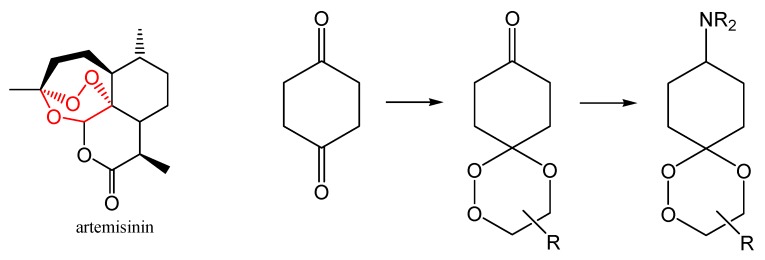


An alternative approach to new peroxides with a 1,2,4-trioxane core is a dimer-route initiated from the peroxyactalization of *vic*-hydroxy hydroperoxides with bifunctional ketones. This approach was stimulated by reports on the improved antimalarial and antitumor activities of dimers of deoxoartemisinin derivatives which have been linked by hydrocarbon chains [11]. In case of *vic*-hydroxy hydroperoxides as peroxide sources, difunctional carbonyl compounds are useful templates for *bis*-peroxyacetalization. Cyclohexane-1,4-dione (CHD) has already been applied as building block for the synthesis of trioxaquines [12] and other amino-substituted antimalarial 1,2,4-trioxanes (Scheme 1) [13].

The peroxyacetalization with the bifunctional CHD can in principle also lead to bis-adducts which have not been reported yet. In the course of our investigations on multifunctional 1,2,4-trioxane syntheses, we have now developed a route to these hitherto unknown structures [10a]. Our interest in these structures was also inspired by recent reports from the Vennerstrom and the O´Neill groups on comparable structures in context of their work on pharmaceutically active tetroxanes, the 6,7,13,14-tetraoxadispiro[5.2.5.2]-hexadecanes [14].

## Results and Discussion

The synthesis of the allylic hydroperoxides **2** from the allylic alcohols **1** proceeded according to literature procedures published by us for solid state photooxygenation [15]. This reaction protocol is advantageous because of the sensitizer-free isolation of the product from the polymer network. The conversion of allylic hydroperoxides into 1,2,4-trioxanes **3** requires Lewis-acid catalysis in the presence of the target carbonyl compound. We have carefully optimized the reaction conditions and in most cases applied the carbonyl compound in excess in order to drive the reaction to completion. In cases where this approach was not possible (e.g. carbonyl compounds with high molecular weight or non-separable substrates) equimolar amounts were used, which drastically reduced the yields of the trioxane products because of Lewis-acid catalyzed hydroperoxide cleavage.

**Scheme 2:**
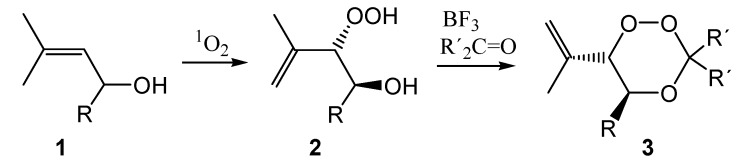


**Scheme 3:**
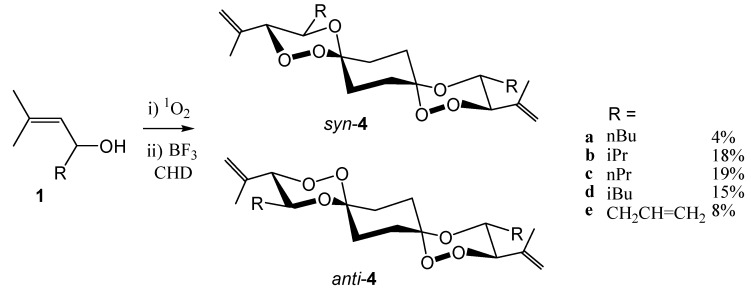


In order to condense allylic hydroperoxides **2** with bifunctional compounds, obviously the hydroperoxides **2** have to be applied in excess. This did, however, additionally increase the competition by the Lewis-acid catalyzed cleavage of the substrate [9c]. The isolated yields of *syn*/*anti*-mixtures of the bis-spiroanellated trioxanes **4** from the reaction with cyclohexane-1,4-dione were consequently low (Scheme 3).

Only products deriving from the *threo*-diastereoisomeric substrates **2** were detected (albeit *threo*/*erythro* mixtures (≈ 8:1) were applied). In the largely preferred low-energy conformation, all substituents at the two trioxane rings are in equatorial the position, simplifying the otherwise complex symmetry of dispiranes [16]. While the basic structures (i.e. the tricyclic ring system **9** – *vide infra* - without substituents) of the *syn*-isomers are of C_1_-symmetry, the *anti*-isomers have inversion symmetry. The two *anti*-isomers **4a** and **4c** were analyzed by X-ray structure analyses. From space-filling models based on several X-ray structures it becomes apparent qualitatively that both dispirane isomers have rod-like core structures decorated with substituents at both ends. In the *syn* spiro compounds the peroxo-bridges are unshielded and exposed to the one side of the dispirane skeleton, whereas in the *anti*-isomers, the 1,2,4-trioxane structures are shielded by the alkyl substituents at the trioxane rings. We are currently studying the consequences on the antimalarial activities of these structural features.

Another substrate which we investigated as a peroxyacetalization substrate was the hydroperoxide **6** derived from the methyl ester of γ-hydroxy tiglic acid [17]. This substrate is a reluctant singlet oxygen acceptor and prolonged irradiation times are necessary. First, we studied the peroxyacetalization of **6** using equimolar amounts of **6** and the monoprotected cyclohexane-1,4-dione **5**. In this case, a mixture of the trioxaspiro[5.5]undecane **7** and the hexaoxadispiro[5.2.5.2]hexadecanes **8** was isolated. The relative proportion of the dispiro product was improved by lowering the amount of the ketone **5** and the yield for **8** could be improved to 20%.

**Scheme 4:**
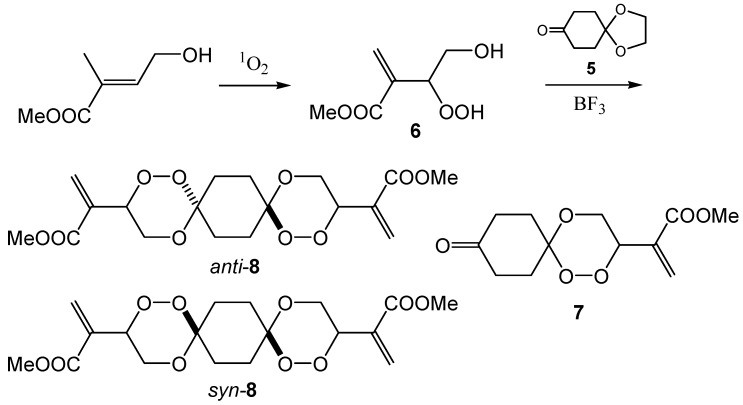


### Structures of 1,2,5,10,11,14-hexaoxadispiro[5.2.5.2]hexadecanes

In all cases, mixtures of *syn*- and *anti*-dispiro[5.2.5.2]hexadecanes were isolated from the *threo*-diastereoisomeric hydroperoxides **2**. Diastereoisomers originating from the *erythro* hydroperoxides and mixed products combing *threo* with *erythro* compounds were formed but could not be isolated in pure forms and were separated from the major products during chromatographic work-up. The X-ray structure analysis of several of these compounds revealed that in all cases all-equatorial conformers exist in the solid state. In liquid phase, analysis of the ^3^J_HH_ coupling pattern (^3^J_HH_ = 9.7 – 10.2 Hz) confirmed that this is also true for isotropic conditions.

The preferred bis-equatorial alignment of the substituents at the stereogenic centers (the former hydroperoxy- and hydroxyl-bearing carbon atoms) restricts the conformational mobility of the 1,2,4-trioxane rings that decorate the central cyclohexane. Only two diastereoisomeric forms were detected in the NMR spectra.

**Scheme 5:**
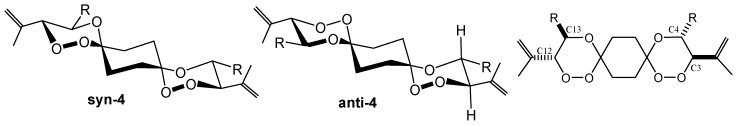


**Scheme 6:**
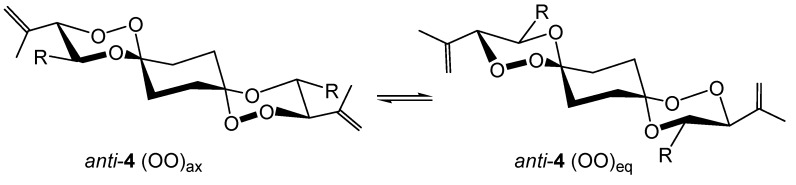


We assume, however, that *syn*-**4** as well as *anti*-**4** exist in two diastereoisomeric forms as well with respect to the relative configuration of the two trioxane ring systems, i.e. *syn*-**4** is a mixture of (3R*, 4R*, 12R*, 13R*) and (3R*, 4R*, 12S*, 13S*) diastereoisomers. Due to the minimal structural differences, these isomers could, however, not be differentiated by spectroscopic methods. Whereas the spirotricyclic ring structures of *syn*-**4** or *syn*-**8** are in a degenerate conformer equilibration with respect to the central cyclohexane, *anti*-**4** or *anti*-**8** can exist in two energetically different conformers, namely the *anti*(OO)_ax_ and *anti*(OO)_eq_ isomers (Scheme 6).

**Figure 1:**
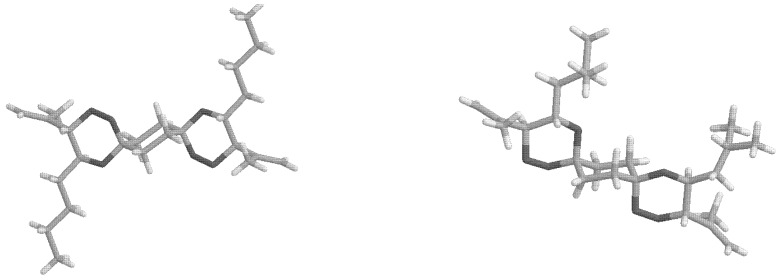
X-ray structure of *anti*-**4a** and *syn*-**4d**.

**Figure 2:**
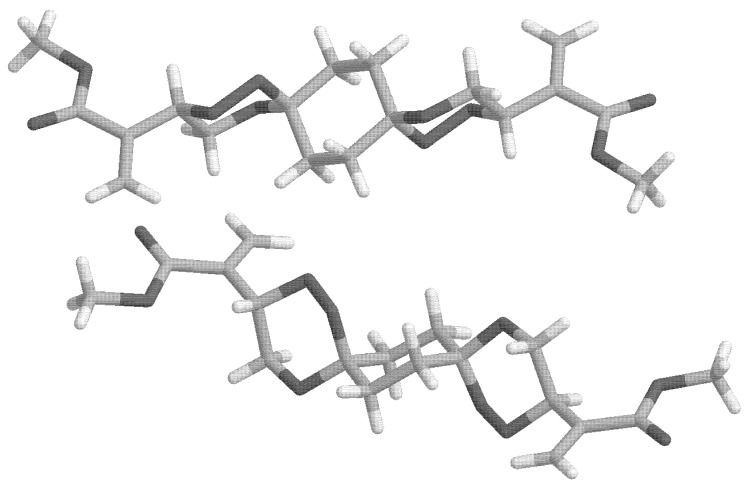
X-ray structure of *anti*-**8** (two chair views).

From the X-ray structure analyses of two of the *bis*-spiro compounds *anti*-**4a** (Figure 1) and compound *anti*-**8** (Figure 2), it appears that the *anti*(OO)_ax_ is preferred in the crystal.

The C-O bond lengths to the spiro carbon atoms are: in *anti*-**4a** C-OO_ax_=1.425 Å and C-O_eq_=1.432 Å, in *syn*-**4d** C-OO_ax_=1.432 Å, C-OO_eq_=1.392 Å and C-O_ax_=1.433 Å, C-O_eq_=1.419 Å, in *anti*-**8** C-OO_ax_=1.436 Å and C-O_eq_=1.437 Å.

### Theoretical Considerations

Beyond the synthetic approach to and chemical features of the new bis-spirofused compounds their structural aspects are of special interest. While the spirotricyclic ring structures of *syn*-**4** are in a degenerate conformer equilibration with respect to the central ring (as shown in Scheme 6), *anti*-**4** can exist in two energetically different conformers, namely the *anti*-**4**(OO)_ax_ and *anti*-**4**(OO)_eq_ isomers. The X-ray structures in Figure 1 and Figure 2 show that the peroxo moieties in each case of an *anti*-structure are situated *axial* with respect to the central cyclohexane ring. In the only exception *syn*-**4d** (Figure 1) one of the peroxo groups is forced into the equatorial position due to structural reasons as long as the central ring is in the chair conformation. 

The axial preference of the peroxo groups in 1,2,5-trioxaspiro[5.5]undecane derivatives also holds for most of the few examples of other compounds which can be found in the Cambridge Structural Database (cf. Table 1). 

**Table 1:** Conformational situation^a^ of the peroxo groups in molecular structures containing a 1,2,5-trioxa-spiro[5.5]undecane substructure.

CSD Structure Code	Sterochemical arrangement of the Peroxo group	Ref.
AWUDUR / AWUFAZ^b^	Axial	18
AWUFED / AWUFIH^b^	axial / equatorial^c^	18
FIXBAQ	Axial	9c
JOJCOA / JOJCUG^b^	axial / equatorial	19

^a^Based on the 3D structures deposited in the CSD Version 5.29 incl. Jan 2008 update. The list is based on structures in which the relative situation of the two rings is not enforced by other structural circumstances like additional bridges etc. and in which the carbon atoms of the trioxacyclohexane ring are in sp^3^-hybridization. ^b^These entries concern the same molecular formula.^ c^This crystal structure contains two different molecular entities, one with an (OO)_ax_ situation the other with (OO)_eq_.

We were interested in investigating whether the predominance of the (OO)_ax_ arrangement allows an educated guess that this molecular arrangement might be preferred due to a stereoelectronic principle. To elucidate this topic in more detail we have started a theoretical study on the basis of density functional theory (DFT) calculations.

All calculations have been performed employing the Becke three parameter hybrid functional [20] with the correlation functional of Lee, Yang and Parr [21] (B3LYP) in combination with the 6-311G(d) basis set as implemented in the Gaussian 03 [22] program under tight convergence criteria and employing an ultrafine integration grid (99,590). The ground state character of each stationary point has been proven by frequency calculations confirming the absence of any negative Eigenvalues in the Hessian matrix. The absolute energies as well as the zero point correction energies (ZPE) have been calculated and compiled in Table 2.

To concentrate on the energetic effects of the (OO)_ax_- vs. (OO)_eq_-alignment in the spirotricyclic ring, i.e. to exclude the significant energetic contribution of the conformational arrangement of the peripheral substituents in **4 ** and **8**, we have calculated and will discuss in the following the common partial structure of all the presented peroxo diacetals **4** and **8**, namely 1,2,5,10,11,14-hexaoxadispiro[5.2.5.2]hexadecane (**9**). For all structures we restrict our considerations on the energetically preferred chair conformation of all the rings, which, of course, is slightly distorted in the case of the trioxane substructures.

**Scheme 7:**
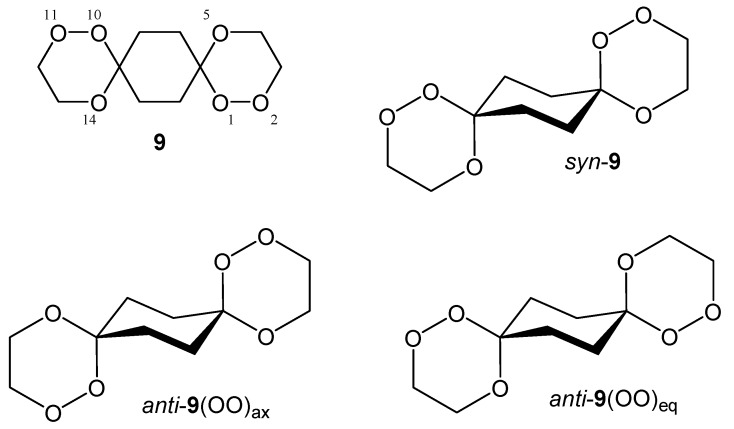


**Figure 3:**
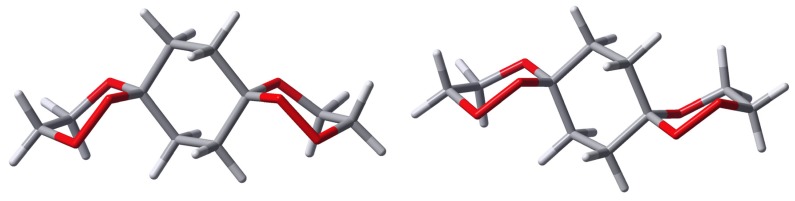
Calculated structures of two distinct conformers (left: equilateral, right: antilateral) of *syn*-**9**.

As already mentioned, *syn*-**9** exists in a degenerate conformational situation with respect to its central cyclohexane ring. Due to the *syn*-arrangement of the peroxo groups, one of them is always located in an axial position while the other is oriented equatorial. This remains so even if the middle ring inverts its chair conformation. Nevertheless, two distinct structures are possible for *syn*-**9** with respect to the relative arrangement of the outer trioxane rings (cf. Figure 3). Both structures are of C_1_-symmetry but in one of them the outer trioxane rings are flipped to the same side of the molecule (equilateral) while in the other they are directed to the opposite sides of the molecule (antilateral), the latter being the energetically favoured.

**Figure 4:**
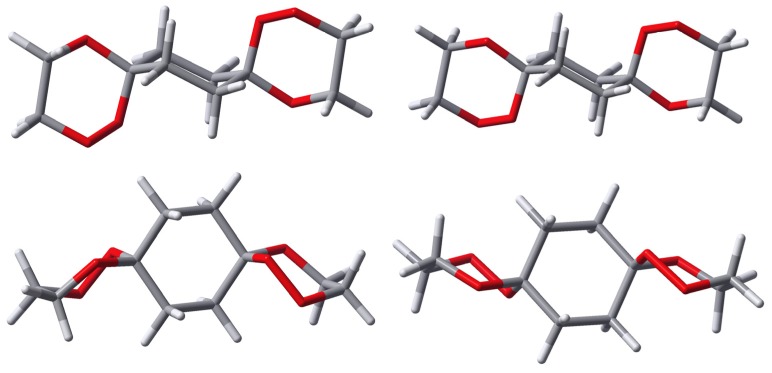
Calculated structures of *anti*-**9**(OO)_ax_, left: equilateral (C_2_), right: antilateral (C_i_). Each conformer is shown from two perspectives (upper and lower part).

Due to the structural arrangement of the peroxo groups *anti*-**9** (cf. Figure 4) their orientation is always the same on both sides, i.e. both are located axial or both are located equatorial, respectively. Each of these middle ring conformers again split up into two distinct conformers with respect to the outer trioxane rings. Here, the equilateral structures possess C_2_-symmetry, while in the antilateral structures are of C_i_-symmetry. The energies calculated for the various different structures of **9** are compiled in Table 2.

**Table 2:** Symmetry and energies^a^ calculated for the model structures **9**.

Structure		Sym.	E_abs._ (Hartree)	E_ZP_ (Hartree)	E_abs._+E_ZP _(Hartree)	E_rel._ (kJ/mol)
*syn*-**9**	antilateral	C_1_	-842.04548417	0.267329	-841.77815517	0.00
*syn*-**9**	equilateral	C_1_	-842.04542546	0.267338	-841.77808746	0.18
*Anti*-**9** (OO)_ax_	antilateral	C_i_	-842.04603154	0.267327	-841.77870454	0.00
*Anti*-**9** (OO)_ax_	equilateral	C_2_	-842.04599155	0.267295	-841.77869655	0.02
*Anti*-**9** (OO)_eq_	antilateral	C_i_	-842.04512847	0.267395	-841.77773347	2.55
*Anti*-**9** (OO)_eq_	equilateral	C_2_	-842.04511732	0.267367	-841.77775032	2.51

^a^E_abs_: absolute SCF energy, E_ZP_: zero point correction energy, E_rel_: relative energy with respect to the energetically lowest conformer.

**Figure 5:**
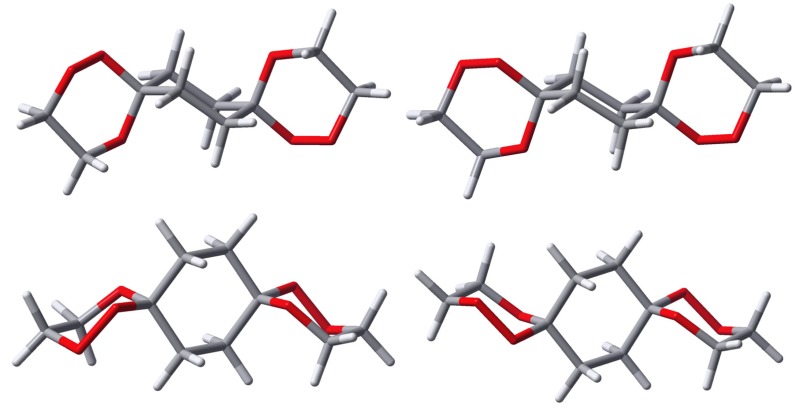
Calculated structures of *anti*-**9**(OO)_eq_, left: equilateral (C_2_), right: antilateral (C_i_). Each conformer is shown from two perspectives (upper and lower part).

As can be seen from the relative energies listed in Table 2, the energetic discrimination between the antilateral and the equilateral arrangement of the outer trioxane rings is negligible. The energetic difference between the (OO)_ax_ and the (OO)_eq_ conformer is still quite small with the axial situation being preferred by about 2.5 kJ/mol. Without putting an overemphasis on this relatively small energy difference (about one-third of a typical anomeric effect) it might be the decisive factor driving the conformational equilibrium towards the (OO)_ax_ arrangement during crystallization – probably supported by intermolecular interactions and packing forces which are not considered in our calculations – and it would not be the first case where nature fells decisions on such small energetic disparities. The origin of the energetic preference of the (OO)_ax_ situation in 1,2,5-trioxa-spiro[5.5]undecane derivatives is the subject of current investigations in our research groups.

## Experimental

### General

IR: Infrared spectra were obtained using Perkin-Elmer 1600 series FTIR spectrometer and are given in cm^-1^ units. Solid samples are measured as CsI or KBr discs while liquids are measured as neat between two NaCl plates; ^1^H-NMR: The ^1^H-NMR spectra were recorded on Bruker AC 300, Bruker DPX 300 spectrometers operating at 300 MHz or on Bruker DRX 500 spectrometer instrument operating at 500 MHz. Chemical shifts are reported as δ in ppm and the coupling constant, *J*, in Hz units. In all spectra solvent peaks were used as internal standard. Solvent used are CDCl_3_ (δ = 7.24 ppm), DMSO-d_6_ (δ = 2.49 ppm), acetone-d_6_ (δ = 2.04 ppm), and MeOH-d_4_ (δ = 3.35, 4.78 ppm). Splitting patterns are designated as follows: s, singlet; d, doublet; t, triplet; q, quartet; m, multiplet; br, broad; ^13^C-NMR: The ^13^C-NMR spectra were recorded either on a Bruker AC 300 spectrometer instrument operating at 75 MHz or on Bruker DRX 500 spectrometer instrument operating at 125 MHz; HRMS: High resolution mass spectra were recorded on Finnigan MAT 900 spectrometer and are measured for the molecular ion peak (M^+^); Combustion analysis: CHN-combustion analyses were measured using Elementar Vario EL Instrument; X-ray analysis: All X-ray measurements were Nonius KappaCCD diffractometer (2Θ_max_ = 54°, MoK_α_ radiation, λ = 0.71073 Å), graphite monochromator, ϕ/ω-scans. The structures were solved using direct methods (SHELXS-97, SHELXL-97).

### General procedure for photooxygenation of allylic alcohols **1**

Commercially available (Aldrich) polystyrene-divinylbenzene copolymer beads (2.5 g) are distributed over a Petri dish (19 cm diameter) and swollen with CH_2_Cl_2_ (20 mL). The substrate (ca. 10 mmol) and the nonpolar sensitizer (TPP – tetraphenylporphyrin - or TTP – tetratolylporphyrin - ca. 3-6 mg) in ethyl acetate (20 mL) are subsequently added and the excess solvent is evaporated by leaving the Petri dish in a well ventilated hood. The Petri dish is then covered with a glass plate and the sandy solid is irradiated with halogen lamp or sodium street lamp. The polymer beads are subsequently rinsed with ethanol (3 x 30 mL) and filtered (the beads are kept for regeneration and reuse). The solvent is evaporated under reduced pressure (caution, water bath temperature should not exceed 30 °C).

### General procedure for the peroxyacetalization reaction used for the synthesis of spiro-bistrioxanes **4**

To a stirred solution of the β-hydroxy hydroperoxide **2** and 0.5 equivalents of cyclohexane-1,4-dione in dry CH_2_Cl_2_ (100 mL) was added at room temperature a catalytic amount of boron trifluoride etherate (ca. 0.2 mL) and the mixture was further stirred for about 12 h (overnight) at the same temperature. The reaction mixture was partitioned between CH_2_Cl_2_ and saturated NaHCO_3_ solution and the phases were separated. The aqueous phase was extracted with CH_2_Cl_2_ (3 x 30 mL) and the combined organic phases were washed with brine, water, dried over Na_2_SO_4_. Solvent evaporation (*Caution*: water bath temperature should not exceed 30 °C!), followed by chromatographic purification afforded the spiro-bistrioxanes as pure products.

### 4,13-Dibutyl-3,12-diisopropenyl-1,2,5,10,11,14-hexaoxadispiro[5.2.5.2]hexadecane (syn-**4a**) and 4,13-dibutyl-3,12-diisopropenyl-1,2,5,10,11,14-hexaoxadispiro-[5.2.5.2]hexadecane (anti-**4a**)

Following the general procedure, a solution of 3-hydroperoxy-2-methyloct-1-en-4-ol (**2a**, 1.45 g, 8.33 mmol) and cyclohexane-1,4-dione (0.46 g, 4.11 mmol) in CH_2_Cl_2_ was treated with a catalytic amount of BF_3_xEt_2_O (0.2 mL). Usual work-up followed by preparative thick-layer chromatography (SiO_2_, EA/*n*-hex, 1:10, R_f_ = 0.53) afforded a 1:1 diastereomeric mixture of the pure 1,2,4-trioxanes *syn*-**4a** and *anti*-**4a** (0.07 g, 0.17 mmol, 4 %) as oil which crystallizes on standing, m.p. 123-124°C; ^1^H-NMR: (300 MHz, CDCl_3_, both diastereomers) δ (ppm) = 0.86 (t, 3H, *J* = 7.0 Hz, CH_3_CH_2_), 1.05-1.72 (m, 8H, CH_2_), 1.60 (m, 3H, CH_3_C=), 2.20 (m, 2H, CH_2_), 3.86 (m, 1H, OCH), 4.25 (d, 1H, *J* = 9.6 Hz, OOCH), 5.04 (s, 2H, CH_2_=); ^13^C-NMR: (75.5 MHz, CDCl_3_, 1^st^ diastereomer) δ (ppm) = 13.9 (q, CH_3_CH_2_), 19.7 (q, CH_3_C=), 22.5 (t, CH_2_CH_3_), 25.2 (t, CH_2_), 27.0 (t, CH_2_CH_2_), 30.4 (t, CH_2_CH_2_), 30.8 (t, CH_2_), 69.8 (d, OCH), 87.7 (d, OOCH), 102.4 (s, OCOO), 118.1 (t, CH_2_=C), 139.2 (s, C=CH_2_); additional significant signals of the 2^nd^ diastereomer δ (ppm) = 19.8 (q, CH_3_C=), 22.5 (t, CH_2_CH_3_), 24.9 (t, CH_2_), 27.1 (t, CH_2_CH_2_), 30.5 (t, CH_2_CH_2_), 31.2 (t, CH_2_), 69.8 (d, OCH), 87.7 (d, OOCH), 102.3 (s, OCOO), 118.1 (t, CH_2_=C), 139.1 (s, C=CH_2_); IR: (Film) ν (cm^-1^) = 3083, 2957, 2873, 1648, 1455, 1373, 1258, 1105, 1007, 928, 911; Elemental Analysis: (C_24_H_40_O_6_, M = 424.57) Calcd.: C 67.89 H 9.50; Found: C 67.43 H 9.37

### 3,12-Diisopropenyl-4,13-diisopropyl-1,2,5,10,11,14-hexa-oxadispiro[5.2.5.2]hexadecane (syn-**4b**) and 3,12-diisopropenyl-4,13-diisopropyl-1,2,5,10,11,14-hexaoxa-dispiro[5.2.5.2]hexadecane (anti-**4b**)

Following the general procedure, a solution of 4-hydroperoxy-2,5-dimethylhex-5-en-3-ol (**2b**, 1.60 g, 10.0 mmol) and cyclohexane-1,4-dione (0.56 g, 5.0 mmol) in CH_2_Cl_2_ was treated with a catalytic amount of BF_3_xEt_2_O (0.2 mL). Usual work-up followed by preparative thick-layer chromatography (SiO_2_, EA/*n*-hex, 1:10, R_f_ = 0.70) afforded a diastereomeric mixture of the pure 1,2,4-trioxanes *syn*-**4b** and *anti*-**4b** in a ratio 1:1 (0.36 g, 0.91 mmol, 18 %) as oil which crystallizes on standing into yellow crystals. ^1^H-NMR: (300 MHz, CDCl_3_, both diastereomers) δ (ppm) = 0.90 (d, 3H, *J* = 6.9 Hz, CH_3_CH), 0.97 (d, 3H, *J* = 6.8 Hz, CH_3_CH), 0.79-1.10 (m, CH_2_), 1.60-1.80 (m, 1H, CH(CH_3_)_2_, 1.74 (m, 3H, CH_3_C=), 3.76 (m, 1H, OCH), 4.45 (d, 1H, *J* = 9.8 Hz, OOCH), 5.07 (m, 2H, CH_2_=); ^13^C-NMR: (75.5 MHz, CDCl_3_, 1^st^ diastereomer) δ (ppm) = 15.0 (q, CH_3_CH), 19.6 (q, CH_3_C=), 19.9 (q, CH_3_CH), 24.8 (t, CH_2_), 28.1 (d, CH(CH_3_)_2_), 31.1 (t, CH_2_), 73.4 (d, OCH), 85.7 (d, OOCH), 102.2 (s, OCOO), 118.0 (t, CH_2_=C), 139.3 (s, C=CH_2_); additional significant signals of 2^nd^ diastereomer δ (ppm) = 20.0 (q, CH_3_CH), 25.0 (t, CH_2_) 30.7 (t, CH_2_); IR: (Film) ν (cm^-1^) = 3083, 2965, 1731, 1649, 1469, 1373, 1258, 1118, 919, 825; Elemental Analysis: (C_22_H_36_O_6_, M = 396.25) Calcd.: C 66.64 H 9.15; Found: C 66.26 H 9.24.

### 3,12-Diisopropenyl-4,13-dipropyl-1,2,5,10,11,14-hexaoxadispiro[5.2.5.2]hexadecane (syn-**4c**) and 3,12-diisopropenyl-4,13-dipropyl-1,2,5,10,11,14-hexaoxadispiro-[5.2.5.2]hexadecane (anti-**4c**)

Following the general procedure, a solution of 4-hydroperoxy-2,5-dimethylhex-5-en-3-ol (**2c**) (1.32 g, 8.25 mmol) and cyclohexane-1,4-dione (0.46 g, 4.11 mmol) in CH_2_Cl_2_ was treated with a catalytic amount of BF_3_xEt_2_O (0.2 mL). Usual work-up followed by preparative thick-layer chromatography (SiO_2_, EA/*n*-hex, 1:10, R_f_ = 0.64) afforded a diastereomeric mixture of the pure 1,2,4-trioxanes *syn*-**4c** and *anti*-**4c** in a ratio 1:1 (0.31 g, 0.78 mmol, 19 %) as oil which crystallizes on standing into yellow crystals, m.p. 103-104°C; ^1^H-NMR: (300 MHz, CDCl_3_, 1^st^ diastereomer) δ (ppm) = 0.88 (t, 3H, *J* = 6.9 Hz, CH_3_CH_2_), 1.26-1.58 (m, 4H, CH_2_CH_2_), 1.67 (m, 2H, CH_2_), 1.72 (m, 3H, CH_3_C=), 2.04-2.32 (m, 2H, CH_2_), 3.89 (m, 1H, OCH), 4.25 (d, 1H, *J* = 9.7 Hz, OOCH), 5.04 (m, 2H, CH_2_=C); ^13^C-NMR: (75.5 MHz, CDCl_3_) δ (ppm) = 13.8 (q, CH_3_CH_2_), 18.1 (t, CH_2_CH_3_), 19.7 (q, CH_3_C=), 25.1 (t, CH_2_), 30.8 (t, CH_2_), 32.9 (t, CH_2_CH_2_), 69.5 (d, OCH), 87.6 (d, OOCH), 102.3 (s, OCOO), 118.0 (t, CH_2_=C), 139.1 (s, C=CH_2_); 2^nd^ diastereomer) δ (ppm) = 13.9 (q, CH_3_CH_2_), 18.1 (t, CH_2_CH_3_), 19.7 (q, CH_3_C=), 24.8 (t, CH_2_), 31.2 (t, CH_2_), 32.9 (t, CH_2_CH_2_), 69.5 (d, OCH), 87.6 (d, OOCH), 102.3 (s, OCOO), 118.0 (t, CH_2_=C), 139.1 (s, C=CH_2_); IR: (Film) ν (cm^-1^) = 3083, 2956, 2873, 1680, 1649, 1454, 1374, 1258, 1105, 1006, 918; Elemental Analysis: (C_22_H_36_O_6_, M = 396.25) Calcd.: C 66.64 H 9.15; Found: C 67.11 H 9.02.

### 4,13-Diisobutyl-3,12-diisopropenyl-1,2,5,10,11,14-hexaoxadispiro[5.2.5.2]hexadecane (syn-**4d**) and 4,13-diisobutyl-3,12-diisopropenyl-1,2,5,10,11,14-hexaoxadispiro-[5.2.5.2]hexadecane (anti-**4d**)

Following the general procedure, a solution of 3-hydroperoxy-2,6-dimethylhept-1-en-4-ol (**2d**) (1.32 g, 7.59 mmol) and cyclohexane-1,4-dione (0.42 g, 3.75 mmol) in CH_2_Cl_2_ was treated with a catalytic amount of BF_3_xEt_2_O (0.2 mL). Usual work-up and further purification of the crude product by preparative thick-layer chromatography (SiO_2_, EA/*n*-hex, 1:10, R_f_ = 0.71) afforded a diastereomeric mixture of the 1,2,4-trioxanes *syn*-**4d** and *anti*-**4d** (0.23 g, 0.54 mmol, 15 %) as oil which crystallizes on standing. ^1^H-NMR: (300 MHz, CDCl_3_, both diastereomers) δ (ppm) = 0.81-0.94 (m, 6H, (CH_3_)_2_CH), 1.06 (m, 1H, CH_2_CH), 1.33 (m, 1H, CH_2_CH), 1.59-1.89 (m, 3H, CH_2_ and CHCH_2_), 1.72 (m, 3H, CH_3_C=), 2.10-2.35 (m, 2H, CH_2_), 3.97 (m, 1H, OCH), 4.24 (d, 1H, *J* = 9.5 Hz, OOCH), 5.05 (m, 2H, CH_2_=C); ^13^C-NMR: (75.5 MHz, CDCl_3_, 1^st^ diastereomer) δ (ppm) = 19.7 (q, CH_3_C=), 21.3 (q, CH_3_CH), 23.5 (d, CHCH_2_), 23.7 (q, CH_3_CH), 25.2 (t, CH_2_), 31.2 (t, CH_2_), 39.6 (t, CH_2_CH), 67.8 (d, OCH), 88.1 (d, OOCH), 102.3 (s, OCOO), 118.2 (t, CH_2_=C), 139.0 (s, C=CH_2_); additional signals of 2^nd^ diastereomer δ (ppm) = 19.6 (q, CH_3_C=), 23.5 (d, CHCH_2_), 24.7 (t, CH_2_), 30.7 (t, CH_2_), 39.6 (t, CH_2_CH), 88.1 (d, OOCH), 102.3 (s, OCOO), 139.0 (s, C=CH_2_); Elemental Analysis: (C_24_H_40_O_6_, M = 424.57) Calcd.: C 67.89 H 9.50; Found: C 67.62 H 9.82.

### 4,13-Diallyl-3,12-diisopropenyl-1,2,5,10,11,14-hexaoxadispiro[5.2 .5.2]hexadecane (syn-**4e**) and 4,13-diallyl-3,12-diisopropenyl-1,2,5,10,11,14-hexaoxadispiro[5.2.5.2]-hexadecane (anti-**4e**)

Following the general procedure, a solution of 3-hydroperoxy-2-methylhepta-1,6-dien-4-ol (**2e**) (1.30 g, 8.23 mmol) and cyclohexane-1,4-dione (0.46 g, 4.11 mmol, 0.5 equiv.) in CH_2_Cl_2_ was treated with a catalytic amount of BF_3_xEt_2_O (0.2 mL). Usual work-up and further purification of the crude product (0.87 g, 2.22 mmol, 54 %) by preparative thick-layer chromatography (SiO_2_, EA/*n*-hex, 1:10, R_f_ = 0.55) afforded a diastereomeric mixture of the pure 1,2,4-trioxanes *syn*-**4e** and *anti*-**4e** (0.13 g, 0.33 mmol, 8 %) as viscous colorless oil. ^1^H-NMR: (300 MHz, CDCl_3_, 1^st ^diastereomer) δ (ppm) = 1.60-2.39 (m, 6H, 3 x CH_2_), 1.74 (s, 3H, CH_3_C=), 3.97 (m, 1H, OCH), 4.31 (d, 1H, *J* = 9.7 Hz, OOCH), 5.07 (m, 4H, CH_2_=CH and CH_2_=C), 5.82 (m, 1H, CH=CH_2_); ^13^C-NMR: (75.5 MHz, CDCl_3_, 1^st ^diastereomer) δ (ppm) = 19.7 (q, CH_3_C=), 24.8 (t, CH_2_), 31.1 (t, CH_2_), 35.3 (t, CH_2_CH=), 69.5 (d, OCH), 86.9 (d, OOCH), 102.4 (s, OCOO), 117.2 (t, CH_2_=CH), 118.4 (t, CH_2_=C), 133.6 (d, CH=CH_2_), 138.8 (s, C=CH_2_); additional significant signals from the 2^nd^ diastereomer δ (ppm) = 3.35 (m, 1H, OCH), 4.30 (d, 1H, *J* = 9.7 Hz, OOCH); ^13^C-NMR: (75.5 MHz, CDCl_3_, 2^nd^ diastereomer) δ (ppm) = 19.7 (q, CH_3_C=), 24.8 (t, CH_2_), 31.1 (t, CH_2_), 35.3 (t, CH_2_CH=), 69.5 (d, OCH), 87.1 (d, OOCH), 102.4 (s, OCOO), 117.1 (t, CH_2_=CH), 118.4 (t, CH_2_=C), 133.7 (d, CH=CH_2_), 138.9 (s, C=CH_2_); Elemental Analysis: (C_22_H_32_O_6_, M = 392.49) Calcd.: C 67.32 H 8.22; Found: C 66.91 H 8.47.

### 2-(9-Oxo-1,2,5-trioxaspiro[5.5]undec-3-yl)acrylic acid methylester (**7**) and 2-[12-(1-methoxy-carbonyl- vinyl)-1,2,5,10,11,14-hexadispiro[5.2.5.2]hexadec-3-yl]acrylic acid methyl ester (syn- and anti-**8**)

Following the general procedure, a solution of 0.33 g (2.0 mmol) methyl 3-hydroperoxy-4-hydroxy-2-methylene-butanoate (**6**) and 0.31 g (2.0 mmol) 1,4‑dioxaspiro[4.5]decan-8-one in CH_2_Cl_2_ (40 mL) was treated with a catalytic amount of p-TosOH (50 mg). Usual work up afforded a mixture of compound **7** and compound **8** (50:50 *anti*/*syn*-diasteroisomeric mixture) in a ratio of 1:1. Following thick-layer chromatography (SiO_2_, EA/petroleum 1:10, R_f_ (**7**)= 0.08, R_f_ (**8**)= 0.12) yielded the pure products as colourless oils, which crystallized on standing. A second experiment was performed using 0.5 eq. of 1,4‑dioxaspiro[4.5]decan-8-one (0.2 g, 1.28 mmol ketone and 0.4 g, 2.55 mmol of the peroxide **6**). In this case a mixture of **7** and **8** was obtained in a ratio of 25:75. Compound **7** was recrystallized from isopropanol/dichloromethane for X-ray analyses. *Compound* **7**: yield: 0.1 g, 0.39 mmol (20 %), m.p.: 83-85 °C; ^1^H-NMR: (CDCl_3_, 300 MHz) δ = 1.98 (m; 2H, CH_2_), 2.06 (m; 2H, CH_2_), 2.43 (m; 4H, CH_2_), 3.77 (s; 3H, CH_3_), 3.89 (dd; 1H, OCH_2_), 4.05 (m; 1H, OCH_2_), 5.21 (dd, *J*_1_ = 9.5 Hz, *J*_2_ = 2.8 Hz; 1H, OOCH), 5.88 (s; 1H, CH_2_ olef.), 6.43 (s; 1H, CH_2_ olef.); ^13^C-NMR: (CDCl_3_, 75 MHz) δ = 33.8 (t; 1C, CH_2_), 36.4 (t; 1C, CH_2_), 36.5 (t; 1C, CH_2_), 38.1 (t; 1C, CH_2_), 52.3 (q; 1C, CH_3_), 64.6 (t; 1C, OCH_2_), 77.1 (d; 1C, OOCH), 101.2 (s; 1C, C_q_), 128.8 (d; 1C, CH_2_ olef.), 135.1 (s; 1C, C_q_ olef.), 164.8 (s; 1C, COOCH_3_), 209.7 (s; 1C, CO); MS: (EI, 70 eV) m/z (%) = 256 (M^+^; < 1), 224 (C_12_H_16_O_4_^+^; 5), 112 (C_6_H_8_O_2_^+^; 70), 57 (C_3_H_5_O^+^; 50), 56 (C_3_H_4_O^+·^; 93), 55 (C_3_H_3_O^+^; 100); IR: (ATR) (cm^-1^) ν = 2957, 2893, 1716, 1631, 1439, 1269, 1115, 1089, 1032, 969, 954, 921, 911. *Compound* **8**: yield: 0.1 g, 0.25 mmol (10 %); ^1^H-NMR: (CDCl_3_, 300 MHz; 1^st^ diastereomer) δ = 1.70 (m; 4H, CH_2_), 1.82 (m; 4H, CH_2_), 3.75 (s; 6H, CH_3_), 3.81 (dd; 2H, OCH_2_), 4.05 (m; 2H, OCH_2_), 5.13 (dd; 2H, OOCH), 5.86 (s; 2H, CH_2_ olef.), 6.40 (s; 2H, CH_2_ olef.); ^13^C-NMR: (CDCl_3_, 75 MHz; 1^st^ diastereomer) δ = 30.6 (t; 4C, CH_2_), 52.2 (q; 2C, CH_3_), 64.3 (t; 2C, OCH_2_), 76.9 (d; 2C, OOCH), 102.0 (s; 2C, C_q_), 128.5 (d; 2C, CH_2_ olef.), 135.3 (s; 2C, C_q_ olef.), 164.9 (s; 2C, CO); additional significant signals of the 2^nd^ diastereomer δ = 30.8 (t; 4C, CH_2_), 64.4 (t; 2C, CH_2_), 102.1 (s; 2C, C_q_), 135.4 (s; 2C, C_q_ olef.); MS: (EI, 70 eV) m/z (%) = 112 (C_6_H_8_O_2_; 100), 57 (C_3_H_5_O; 20), 56 (C_3_H_4_O; 33), 55 (C_3_H_3_O; 52); IR: (ATR) ν (cm^-1^) = 2956, 2853, 1725, 1634, 1438, 1273, 1250, 1199, 1161, 1116, 967, 954, 920, 814.
